# Sheathless guiding catheter from a femoral approach for complex percutaneous coronary interventions —An unusual solution for an often‐encountered problem

**DOI:** 10.1002/ccr3.2540

**Published:** 2019-11-22

**Authors:** Zeev Israeli, Irina Nordkin, Alexander Goldberg, Majdi Halabi

**Affiliations:** ^1^ Ziv Medical Center Affiliated With the Faculty of Medicine in Zefat Bar Ilan University Tzfat Israel

**Keywords:** complex percutaneous coronary interventions, rotational atherectomy, sheathless catheter

## Abstract

Sheathless guiding catheters are a valuable tool in the catheterization labor and may assist the operator when dealing with heavily calcified and tortuous vessels. Nevertheless, when hostile anatomy prevents successful percutaneous coronary interventions (PCI) from the radial access, transfemoral use of sheathless guide can assist in overcoming these challenges in a safe manner.

## Graphical abstract

Sheathless guiding catheters are a valuable tool in the catheterization labor and may assist the operator when dealing with heavily calcified and tortuous vessels. Nevertheless, when hostile anatomy prevents successful PCI from the radial access, transfemoral use of sheathless guide can assist in overcoming these challenges in a safe manner.

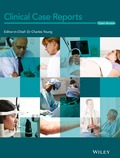

## 1 INTRODUCTION

As the population grows older and patients live longer with multiple morbidities, the care for these individuals is becoming increasingly challenging. Interventional cardiologists are facing ever‐growing complexity of coronary and peripheral vascular disease which demands high quality, specially engineered equipment and material for successful percutaneous coronary interventions (PCI). This includes negotiating with small, calcified, and tortuous vessels that may often hinder the use of large, supportive guiding catheters.

Sheathless guiding catheters (GC) are an example of such a device. These catheters are designed for the “radial era,” as they minimize radial puncture site while providing a large inner lumen. Additionally, the hydrophilic coating enhances their trackability through tortuous vessels. A wide spectrum of sizes and shapes is available, allowing coronary intubation of all anatomical varieties. Current data support the use of these catheters for complex PCI including rotational atherectomy.^1^, ^2^

We report, for the first time to the best of our knowledge, about the use of a sheathless GC from a femoral approach for rotational atherectomy.

## 2 CASE HISTORY

This is a case of an 83‐year‐old patient arriving for a planned PCI to his left anterior descending (LAD) artery. Based on the findings of a previous angiogram (in which PCI to his right coronary was completed), we planned to complete rotational atherectomy to the calcified LAD prior to stent implantation. The patient's medical history includes chronic renal failure on dialysis, diabetes, pacemaker implantation (Micra™, Medtronic), hypertension, and hypercholesterolemia. Though current guidelines recommend consideration of coronary artery bypass grafting for diabetic patients with multiple vessel disease including the LAD, the patient was not considered to be a candidate for bypass surgery. His old age, fragileness, as well as the heavily calcified aorta (“porcelain aorta”), made surgical revascularization a nonviable option for him.

A former PCI was undertaken from the right radial artery. However, significant calcification of peripheral vessels including the radial artery prevented a full insertion of the radial sheath into place and the patient suffered severe local pain.

Therefore, for the current procedure, right ulnar access was planned (the patient has an arteriovenous shunt in the left arm). Following the draping of the right wrist in the usual manner, a 6F sheath was inserted into the ulnar artery. Again, significant calcification of his peripheral vessels (video S1) and aorta prevented full insertion of a sheath or the use of any catheter other than a diagnostic 4F right Judkin (several 6F GC, as well as a sheathless, were tried). For completion of the procedure, alternative access in the right femoral artery was placed. Nevertheless, we encountered similar issues while trying to advance multiple GC through a heavily calcified aortic arch. Hence, a PB‐3.5 7.5F SheathLess Eaucath (Asahi Intec) was used. This guiding catheter has a large inner diameter of 2.06 mm and an outer diameter of 2.49 mm. The catheter entails two different braiding patters to provide for optimal torque and flexibility and a hydrophilic coating for improved trackability. The introducer sheath in the femoral artery was exchanged over a standard J‐tipped 0.035‐inch wire for the PB‐3.5 guiding catheter which was easily negotiated into the ascending aorta. Then, the guide's central dilator was removed, followed by left coronary intubation in the usual manner. A Rotawire™ (Boston Scientific) was manipulated to cross the calcified lesion in the mid LAD, and two “runs” of a 1.5 mm burr (burr speed was set to 180 000 rounds per minute and intracoronary infusion of a “cocktail” containing nitroglycerine, verapamil, and heparin given) were completed (video S2). Several balloon inflations followed for effective calcium “cracking” before a 3.5x24 mm EluNIR stent (Medinol) was placed and final postdilation with a 3.75 × 20 mm Accuforce^®^ non‐ compliant balloon (Terumo) provided an excellent result. During PCI, heparin was given for anticoagulation and activated clotting time (ACT) was maintained over 250 seconds. Upon completion of the procedure, ulnar access was removed while maintaining hemostasis with a TR Band^®^ radial artery compression device (Terumo^®^). The guiding catheter was exerted over the J‐ tipped wire while applying local pressure on the groin and exchanged with a femoral 6F sheath (AVANTI+^®^, Cordis^®^). Removal of the femoral sheath was undertaken hours later once ACT dropped below 160 seconds and manual compression was applied. The patient was discharged well the next morning.

## 3 DISCUSSION

The current case represents a unique solution for an often‐encountered problem—torturous and calcified vessels limiting the use of supportive large‐caliber catheters. In extreme cases, such as heavily calcified peripheral vessels, the use of a sheathless catheter from transfemoral access may be the perfect combination of “both worlds”—the larger diameter of the femoral artery with exceptional characteristics of the sheathless catheter designed for the radial artery.

Transradial access route has become the preferred approach due to its proven benefits including a reduction in vascular complications, bleedings, as well as the patient's comfort and earlier mobilization. Most interventional procedures are currently undertaken with the use of a 6F sheath and guiding catheters. These systems are compatible with most interventional techniques and devices, allow completion of complex PCI, and are well tolerated by patients. Nevertheless, the sheath outer diameter is 2F larger than the corresponding guide catheter. With a mean radial artery diameter of 2.4mm ± 0.5,^3^ it is clear that for many patients a 6F sheath is too big.^3^ Local artery injury at the site of sheath insertion is common—an OCT‐based work showed local tears or even intima dissection.^4^ This local phenomenon may lead later to the formation of neointima with a reduction of the artery inner lumen 5 and increase the chances of failure of repeat catheterization via the radial artery.^6^ Moreover, local pain sensation, as well as the risk of radial artery occlusion/slow flows, increases when sheath size exceeds radial diameter.^7^, ^8^

One of the options to minimize radial injury is the advancement of a guide catheter over a dilator without using a sheath. The Asahi EuCath system was introduced in Japan 15 years ago and later received European and American approval.^3^ The catheters are double‐braided to optimize torque and flexibility and are hydrophilic coated. Usually, the operator would initially place a small sheath in the artery for completion of the diagnostic procedure. Next, for coronary intervention, the sheath is withdrawn over a 0.035‐inch J‐tipped wire and the sheathless GC and dilator are introduced. Several sizes are available (6.5,7.5 and 8.5F) as well as multiple curves to fit the patient's anatomy. Thus, the 6.5F sheathless catheter has an inner lumen of a regular 6F guide but an outer diameter that is lower than a 5F sheath. A Canadian trial showed that the use of the Asahi sheathless catheter for PCI was associated with easier arm navigation and less patient discomfort when compared with standard GC.^9^

Though they offer several advantages, the catheters are stiffer than the standard and their advancement and manipulation may be more challenging. Furthermore, the exchange of the sheath for the sheathless system necessitates two men's work since local pressure on the access site is required to prevent excessive bleeding. Lack of sheath to lock the catheter in place and its hydrophilic coating may limit its backup support. Moreover, when radial artery intima‐media thickness (IMT) was measured acutely post PCI in patients that have undergone PCI with a 7F sheathless vs regular 6F GC, IMT was similar in both groups.^10^ Likewise, 90 days later, radial artery occlusion and IMT were alike for both catheter types.^10^ Therefore, it seems that sheathless guides may not provide excessive protection for the radial artery.

Other options for dealing with small peripheral arteries exist. Glidesheath Slender^®^ (Terumo) offers the smallest outer diameter while performing diagnostic or interventional procedures. The thin‐wall design reduces the outer diameter by 1F while maintaining a large inner lumen. They are available in 5,6, and 7F and are of particular advantage used in women or patients with small radial arteries. Nevertheless, the Slenders’ length is limited (10 or 16 cm long) and would not have assisted us in navigating the catheters through the patient's heavily calcified aorta. Long sheaths may also assist while tackling similar issues, but are not widely available.

The current case demonstrates a unique challenge due to the patient's anatomy. Not only were his right arm's vessels tortuous and calcified, but his aorta as well. Though sheathless GC is often helpful when tackling those challenges, we could not complete the procedure from the wrist. The very same problems hindered the use of regular GC from a transfemoral approach. Thus, the practice of sheathless GC from the groin came to use, as the patient, in our opinion, had no other options. The catheter's unique design including a hydrophilic coating, central dilator that eliminates the transition between the wire and GC (thereby “smoothing” its passage in calcified vessels) and the larger diameter of the femoral artery all contributed to our success while facing hostile anatomy.

Yet, the lack of evidence to support the technique should possibly limit its use. For most patients with similar anatomy, a radial‐first approach is reasonable, with tools such as Glidesheath Slender^®^ or sheathless GC when needed. Nevertheless, operators should bear in mind that when thought‐provoking cases are encountered and the “usual tricks” are just not good enough; sheathless GC use from the femoral artery can transform a frustrating procedure to a successful one.

## 4 CONCLUSION

The use of a sheathless GC from the transfemoral approach could offer an exceptional solution for patients with heavily calcified vessels in which successful PCI cannot be completed through radial access.

## Conflict of Interest

None declared.

## AUTHOR CONTRIBUTIONS

ZI and MH: preformed the procedure and wrote the manuscript; IN and AG: revised the manuscript.
